# Modulation of the tumour microenvironment in hepatocellular carcinoma by tyrosine kinase inhibitors: from modulation to combination therapy targeting the microenvironment

**DOI:** 10.1186/s12935-021-02435-4

**Published:** 2022-02-11

**Authors:** Ruyin Chen, Qiong Li, Shuaishuai Xu, Chanqi Ye, Tian Tian, Qi Jiang, Jianzhen Shan, Jian Ruan

**Affiliations:** Department of Medical Oncology, Key Laboratory of Cancer Prevention and Intervention, The First Affiliated Hospital, Zhejiang University School of Medicine, Ministry of Education, Hangzhou, Zhejiang China

**Keywords:** Hepatocellular carcinoma, Tyrosine kinase inhibitors, Tumour microenvironment, Combination therapy, Epithelial–mesenchymal transition

## Abstract

Hepatocellular carcinoma (HCC) is the third leading cause of cancer deaths worldwide. Tyrosine kinase inhibitors (TKIs) remain the backbone of systematic therapy for advanced hepatocellular carcinoma. Sorafenib and lenvatinib are currently approved as first-line therapeutic drugs, and regorafenib and cabozantinib are applied as second-line treatments. With inhibition of angiogenesis as the main target, TKIs exert a profound effect on the tumour microenvironment (TME). The TME is a complex mixture of cellular and noncellular components surrounding the tumour mass, and is associated with tumour progression partially through the epithelial–mesenchymal transition. Specifically, the TME of HCC is characterized by profound extracellular matrix remodelling and an immunosuppressive microenvironment. The purpose of this review is to provide a summary of TME remodelling mediated by four Food and Drug Administration approved TKIs in HCC and thus summarize the rationale and potential targets for combination therapy. The modulatory effect of TKIs on the TME of HCC was reported to enhance the antitumour effect of TKIs through pyroptosis of macrophages and subsequent natural killer cell activation, T cell activation, regulatory T cell reduction in HCC. Meanwhile, TKIs also induce drug resistance via M2 polarization and accumulation, recruitment of tumour-associated neutrophils, and induction of the epithelial–mesenchymal transition. In conclusion, the effect of TKIs on TME can enhance its antitumour effect, but might also partially contribute to the drug resistance that hinders the progression of TKIs as treatment for HCC. Additionally, the effect of TKIs also provides the rationale for combination therapy, including combining TKIs with immune checkpoint inhibitors, to facilitate increased drug efficacy of TKIs.

**Supplementary Information:**

The online version contains supplementary material available at 10.1186/s12935-021-02435-4.

## Background

Hepatocellular carcinoma (HCC) remains the third leading cause of cancer deaths, but the mechanisms underlying tumour initiation and progression are not yet fully understood [1, 2]. Current therapy for HCC mainly includes surgery and liver transplantation, percutaneous ablation, transcatheter arterial chemoembolization, and systematic treatment. Although surgery and liver transplantation are curative treatment options producing significant survival benefits, they can only be applied to the well-selected candidates [3, 4]. In addition, HCC is usually diagnosed at an advanced stage due to rapid progression and a high metastatic rate [5, 6]. Consideration of comorbidities, functional status, and tumour burden limit the use of resection, liver transplantation, percutaneous ablation and transcatheter arterial chemoembolization [7]. In this context, the development of systematic therapy for patients with advanced-stage HCC is urgently needed.

**Fig. 1:**
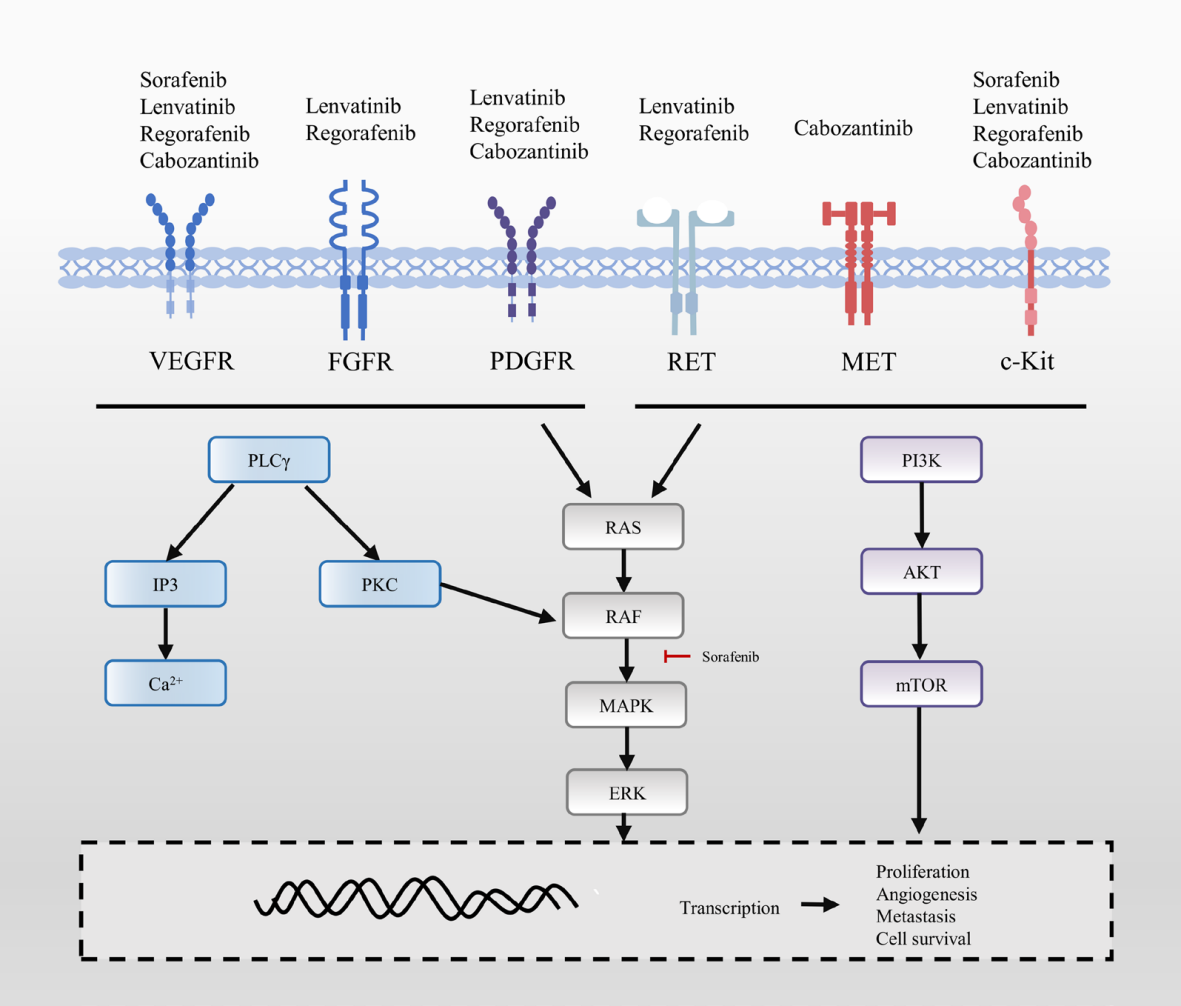
Elucidation of the mechanism of action of TKIs. Sorafenib, lenvatinib, regorafenib, and lenvatinib exert the antitumour activity through blockage of specific targets, subsequently inhibiting of subsequent signalling pathways and eventually inhibiting the proliferation, angiogenesis, migration and survival of tumour cells

The application of multitarget tyrosine kinase inhibitors (TKIs) is significant progress in the systematic treatment of HCC, especially for patients with advanced disease [8]. TKIs bind to tyrosine kinase receptors and activate intracellular signalling via autophosphorylation of the cytoplasmic domains, leading to subsequent antiangiogenic effects [9]. Tumorigenesis is associated with genetic dysregulation involved in a variety of processes, including angiogenesis [10]. Astonishing outpouring of evidence over the previous 50 years has shown that tumour angiogenesis is required for tumour progression and proliferation [11–13]. Tumour angiogenesis has been revealed as an uncontrollable and unorganized process with a balance shifting towards a proangiogenic milieu to maintain angiogenesis [14, 15]. Multiple proangiogenic drivers are involved in angiogenesis, including vascular endothelial growth factor (VEGF), fibroblast growth factor (FGF)-2, platelet-derived growth factor (PDGF), transforming growth factors (TGFs)-beta and alpha, epidermal growth factors (EGF), angiopoietins, and hypoxia-inducible factor (HIF)-1 [15]. Among them, VEGF has been shown to be essential for angiogenesis [16]. To date, the Food and Drug Administration (FDA) has approved four TKIs including sorafenib, lenvatinib, regorafenib, and cabozantinib [17–21]. Sorafenib and lenvatinib are approved as first-line treatments, and cabozantinib and regorafenib are applied as second-line treatments [9, 22]. The details of other TKIs undergoing clinical trials for advanced HCC patients as single agents are also presented (Table 1). Inhibition of vascular endothelial growth factor receptors (VEGFRs) serves as the common mechanism of TKIs, while each TKI possesses a unique profile (Fig. 1) [23].

**Table 1:** Ongoing clinical trials of other TKIs as single agents for HCC

Trial	Identifier	Study arm	Phase	Enrolment	Primary endpoint	State
N/A	NCT02508467	Fisogatinib (BLU-554)	1	150	Safety	Active, not recruiting
NTAHCC	NCT03950518	Anlotinib Hydrochloride; Anlotinib Hydrochloride Capsules; Anlotinib;Levamisole	3	300	PFS	Recruiting
N/A	NCT04985136	Camrelizumab + rivoceranib; sorafenib; regorafenib; rivoceranib	3	482	Stage I: ORR; stage II: OS	Enrolling by invitation
N/A	NCT03945799	Anlotinib hydrochloride	1/2	60	DFS	Recruiting
N/A	NCT01737827	INC280	2	38	Time to progression per RECIST 1.1	Active, not recruiting
JAKaL	NCT04358185	Itacitinib	1	25	AE; ORR	Recruiting
N/A	NCT03582618	Foslinanib + sorafenib	2	40	ORR	Active, not recruiting
N/A	NCT03941873	Sitravatinib; sitravatinib + tislelizumab	1/2	111	AE; SAE per NCI-CTCAE 5.0	Active, not recruiting
AFHC	NCT04954521	Anlotinib	N/A	200	PFS	Recruiting
N/A	NCT05070247	TAK-500	1	106	TEAE grades per NCI CTCAE 5.0; AE; SAE	Not yet recruiting
N/A	NCT03195699	TTI-101	1	60	Safety	Recruiting

*PFS* progression free survival, *DFS* disease free survival, *RECIST* response evaluation criteria in solid tumours, *CTCAE* common terminology criteria for adverse events, *TEAT* treatment emergent adverse events, *NCI* national cancer institute, *AE* adverse events, *SAE* serious adverse events

Despite the promising future of TKIs shown in trials, the clinical outcomes of TKIs are limited, secondary to the high rate of drug resistance [24, 25]. Therefore, the mechanisms underlying the progression and metastasis of HCC still require investigation. The tumour microenvironment (TME) has been shown to be associated with drug resistance, indicating that the modification of tumour cells may be insufficient to improve therapeutic efficacy [26]. The TME is defined as a complex mixture of cellular and noncellular compartments surrounding tumour mass that plays a pivotal role in hepatocarcinogenesis, tumour invasion, and tumour metastasis partially through the epithelial–mesenchymal transition (EMT) [27, 28]. The cellular compartment includes hepatic stellate cells, fibroblasts, immune cells, and endothelial cells, while the noncellular compartment consists of extracellular matrix proteins, growth factors, proteolytic enzymes, and inflammatory cytokines [28]. Specifically, the tumour microenvironment of HCC has unique characteristics including extensive extracellular matrix and immunosuppressive microenvironment [29–31]. The TME was shown to account for the therapeutic effect of TKIs in HCC, but might also induce resistance attenuating the efficacy. For instance, sorafenib was shown to activate natural killer (NK) cells and promote tumour regression, whereas other researchers observed the induction of immunosuppression through increasing the number of regulatory T (Treg) cells and Tumour-associated neutrophils (TANs), as well as subsequent EMT induction leading to drug resistance [32–34]. Summarizing the effects of TKIs on the TME of HCC may assist in improving our understanding of the resistance-inducing mechanism and antitumour effect of TKIs.

Additionally, since TKI monotherapy showed limited clinical benefits, combination therapy has been extensively investigated to counteract the intrinsic resistance induced by TKIs through enhancing the therapeutic efficacy. The representative combination therapy is TKIs and immunotherapy. The past decade has witnessed the development of immunotherapy for HCC. Anti-programmed cell death ligand 1 (anti-PD-1) antibodies have generated impressive outcomes and have been approved as second-line therapies, but the clinical benefits generated are limited due to a low response rate and drug resistance. In patients with advanced HCC, nivolumab, an anti-PD-1 agent, produced an objective response rate (ORR) of 20% in the dose expansion phase of a phase 1/2 clinical trial treating 214 patients with 3 mg/kg nivolumab. Nineteen percent of patients were reported to present grade 3/4 treatment-related adverse events, indicating a manageable safety profile of nivolumab [35]. Another anti-PD-L1 agent, pembrolizumab produced an ORR of 17% in the phase 1/2 Keynote-224 trial that treated 104 eligible patients and was reported to induce serious adverse effects in 15% of patients, with the most common adverse effect being increased level of aspartate aminotransferase (7%) [36]. The latest progress has shed light on combination therapy to increase the ORR and overall survival (OS) of immune checkpoint inhibitors (ICI) in advanced HCC. The combination of atezolizumab and an anti-VEGF antibody, bevacizumab was recently approved by the FDA as first-line therapy for unresectable HCC based on an improved progress-free survival and a superior OS [37]. The updated data has revealed a median overall survival of 15.6 months in the Atezo + Bev arm compared to 13.4 months in the sorafenib arm (hazard ratio 0.66, 95% confidence intervals 0.52–0.85; *P* = 0.0009) and a superior ORR (29.8%) [38]. Based on observations of the immunomodulatory effect of TKSs on the TME, investigations of the combination of TKIs with immunotherapy have become one of the future directions [39–41].

Hence, a deeper understanding of the correlation between TKIs and the TME enables the elucidation of the mechanism underlying the antitumour effect of TKIs on HCC, and possibly provides insights into targets that may reduce drug resistance. In this review, we described the features of the TME of HCC, indicating its role in tumour progression, and then summarized the TME remodelling effect induced by each TKI in detail. Additionally, the rationale for and current state of the combination of TKIs with anti-PD1 antibodies were highlighted in this part to provide an integrated understanding. We also discussed the progress in therapeutic approaches to combine TKIs with other agents related to TKI-induced TME remodelling. Furthermore, we provided insights into future directions.

## The TME of HCC and its role in tumour progression

**Fig. 2:**
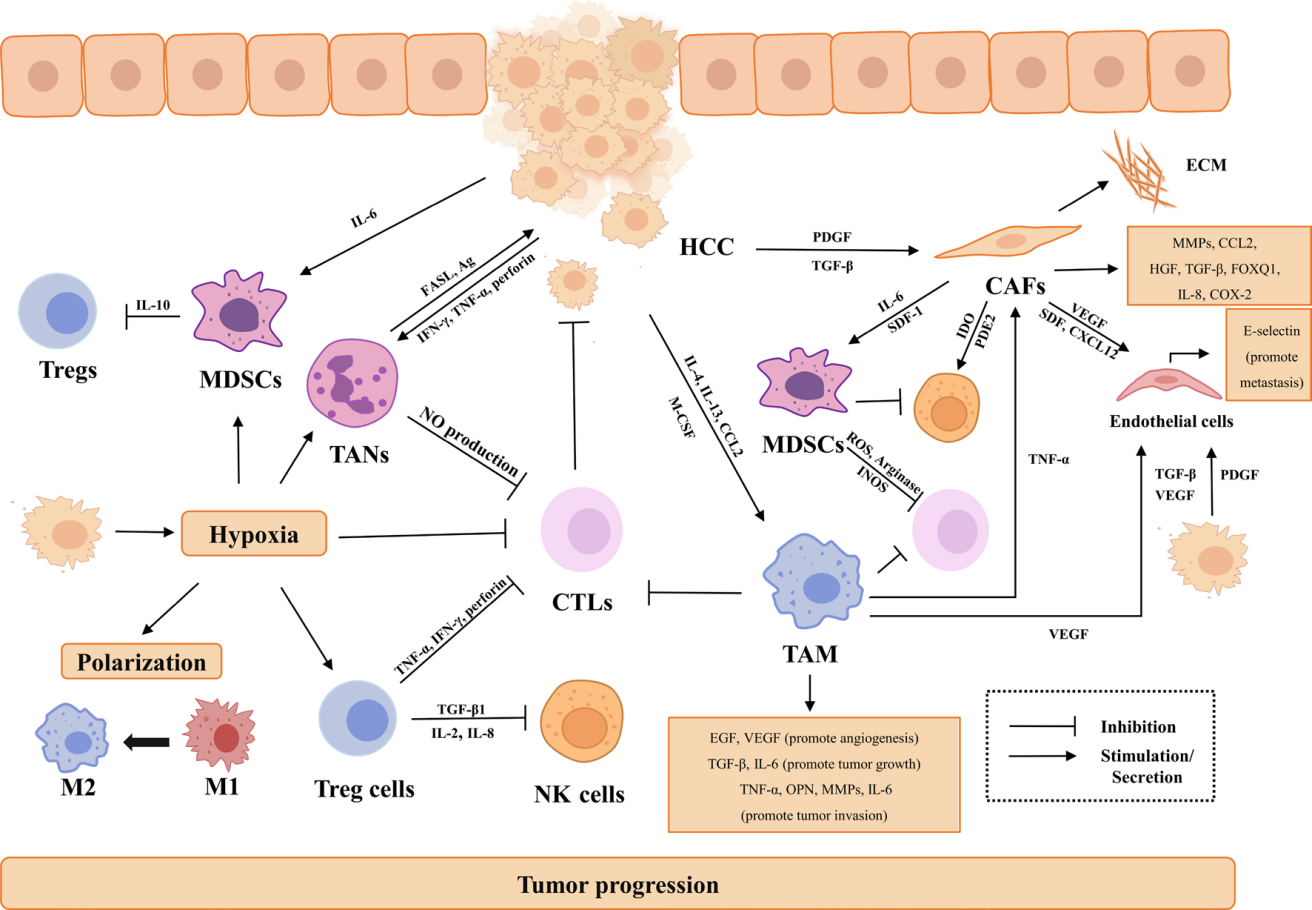
Cell–cell interactions within the TME of HCC that promote tumour progression. The TME of HCC is composed of diverse cellular compartments that play roles in tumour progression through cellular interactions. Suppressive immune subsets infiltration, including MDSCs, TAMs, and Tregs and cellular interaction contribute to immune evasion. The nonimmune compartment, including CAFs and endothelial cells, forms a dynamic network with immune cells, which significantly amplifies the progression of HCC. Among the molecules secreted by CAFs, MMPs, FOXQ1, and CCL2 are associated with tumour metastasis. HGF is associated with EMT, tumour metastasis, migration, and invasion. TGF-β is suggested to play an immunosuppressive role, promote tumour proliferation, and regulate EMT. IL-8 is shown to promote HCC invasion. COX-2 is indicated to contribute to tumour angiogenesis and tumour cell proliferation

Distinguished from other malignancies, the TME of HCC is characterized by profound extracellular matrix remodelling and immunosuppressive microenvironment [29–31]. As the inflammatory state contributes significantly to tumorigenesis in HCC, the immune infiltration in HCC is abundant. NK cells and cytotoxic T cells are involved in the cytotoxic effect [42]. The level of lymphocyte infiltration is associated with recurrence of HCC following liver transplantation [43]. However, other abundant immunosuppressive cells including tumour-associated macrophages (TAMs), Tregs, and myeloid-derived suppressive cells (MDSCs) lead to immune evasion. The nonimmune compartment, including cancer-associated fibroblasts (CAFs) and endothelial cells, forms a dynamic network with immune cells, which significantly amplifies the progression and chemoresistance of HCC through the induction of an immunosuppressive microenvironment (Fig. 2) [44–46]. Additionally, inhibitory programmed cell death protein 1 (PD-L1) molecules and PD-1 upregulation on CD8+ cytotoxic cells are observed in patients with HCC [47, 48]. Characteristics of the TME in HCC provide significance for investigating TME remodelling caused by therapy.

## TKIs-induced TME remodeling

We have searched the PubMed and Web of Science databases, and the strategies were provided (Additional file 1: Fig. S1). TME remodeling triggered by TKIs was categorized according to the remodelled immune cells. The subsequent effect on the EMT was also summarized. As the interaction between TKIs and TME provides a rationale for their combination with immunotherapy, including anti-PD-1 therapy, the rationale and current stage will also be mentioned in this section to provide a better understanding.

### Sorafenib

Since no effective therapy for advanced HCC was available before its application, sorafenib is regarded as the breakthrough point in the therapy of advanced HCC [24]. It has been approved by the FDA based on the results of a phase 3, double-blind clinical trial that involved 602 participants and generated a 3 month longer median survival (hazard ratio 0.69, 95% confidence intervals 0.55–0.87; *P* < 0.001) (NCT00105443) [24]. Sorafenib achieves its therapeutic effect by suppressing angiogenesis mainly through the inhibition of VEGFR1, 2, and 3. Meanwhile, it also inhibits the Ras/Raf/MEK/ERK signalling pathways, PDGFR-β, and hepatocyte factor receptor (c-KIT) (Fig. 1) [49]. The adverse effects of sorafenib include constitutional symptoms, dermatological events, gastrointestinal symptoms, liver dysfunction, and pain [24, 50]. The most frequent side effects observed in the clinic are diarrhoea and hand-foot skin reaction [18, 24, 50]. In addition to the aforementioned effects, an understanding of its effect on TME modulation might provide some extra insights for future research.

**Fig. 3:**
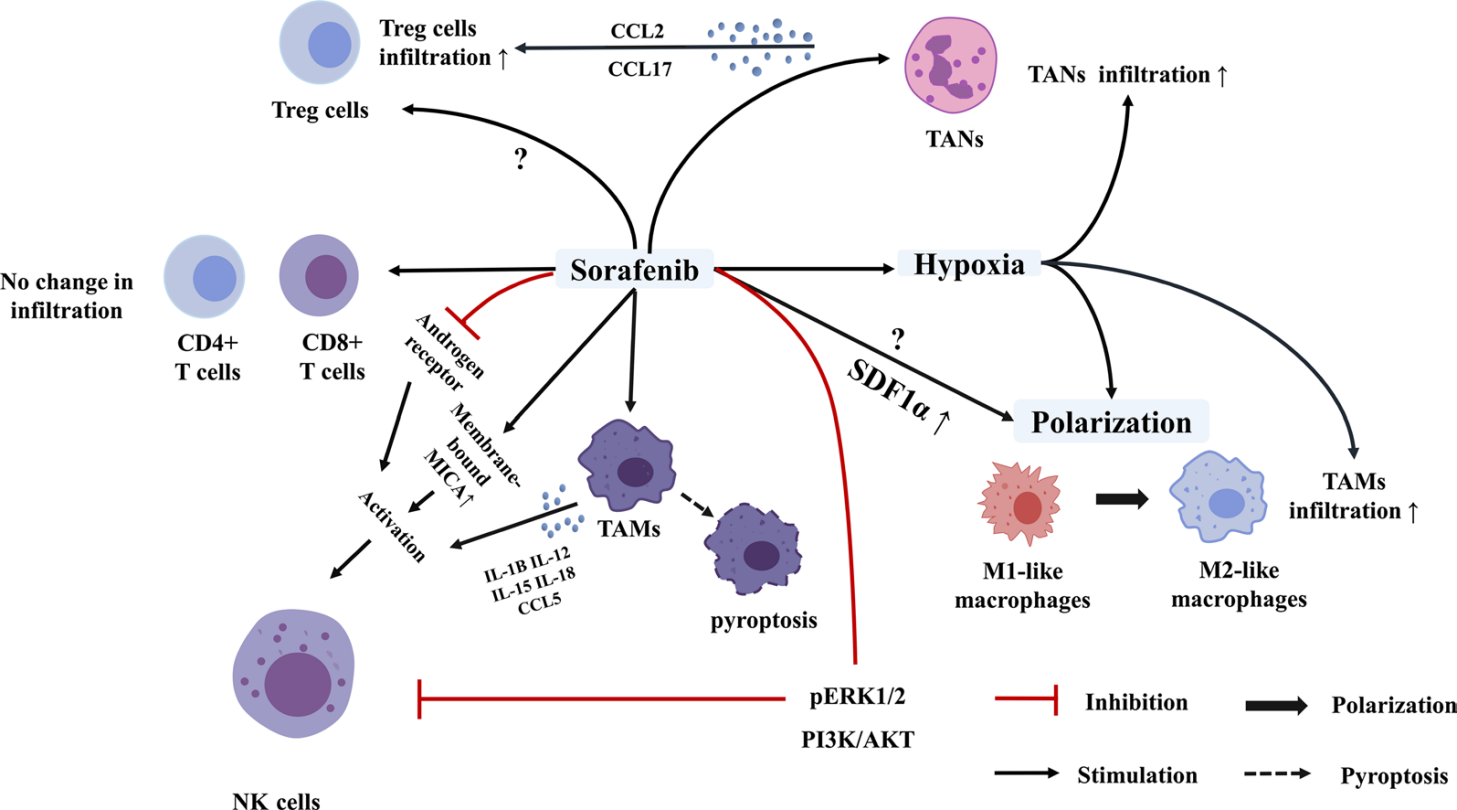
Immunomodulatory effect of sorafenib on HCC. Sorafenib was shown to activate NK cells by regulating the shedding of major histocompatibility complex class I-related chain A (MICA), interacting with macrophages, and inhibiting androgen receptor, but was also shown to inhibit NK cell proliferation through the pERK1/2 pathway. Its effect on altering macrophage polarization from M2 to M1 remains controversial. CD4+ and CD8+ T cell infiltration remained unchanged after sorafenib treatment. Moreover, sorafenib was shown to increase the numbers of TANs and the levels of CCL2 and CCL17, leading to a subsequent increase in Treg cell infiltration, but other studies reported that sorafenib inhibited the recruitment of Treg cells in HCC. TAM polarization and increased infiltration of TAMs and TANs were proposed to be induced by hypoxia after sorafenib treatment

Sorafenib has been reported to interact with immune cells and induce an immunomodulatory effect, yet the modulatory effect of sorafenib remains to be comprehensively clarified (Fig. 3). Among them, the interaction between sorafenib and NK cells was proposed to be significant. NK cells serve as the key effectors in tumour immunosurveillance. Strategies targeting NK cells include natural-killer group 2, member D (NKG2D) ligands, NK cell engagers, NKG2A, and adoptive NK cell strategies [51]. Among them, NKG2D is expressed by NK cells and activates subsequent cytotoxic lymphocytes when binding to ligands, including major histocompatibility complex class I chain-related protein A and B (MICA, and MICB), on target cells [52]. Shedding of endogenous MICA and MICB, which plays an important role in immune evasion, largely depends on a disintegrin and metalloproteinase 9, 10 and 17 (ADAM9, ADAM10, and ADAM17), as well as matrix metalloprotease (MMP) 14 [53–55].

Sorafenib was reported to activate NK cells mainly through regulating the shedding of MICA, interacting with macrophages, and inhibiting androgen receptors. Sorafenib could inhibit the expression of ADAM9, a metalloproteinase that was shown to inhibit the shedding of MICA, subsequently increasing the NK sensitivity of HCC cells [56]. Activation of NK cells following sorafenib treatment was also suggested to be associated with the interaction between macrophages and NK cells. Sorafenib administration activated NK cells in C57BL/6 wildtype and tumour-bearing mice. In an in vitro model, NK cells were activated by sorafenib-treated macrophages in a dose-dependent manner and showed increased degranulation and secretion of interferon (IFN)-γ. The activation of NK cells by sorafenib-treated macrophages was shown to depend on nuclear factor-kappa B (NF-κB), IL12, and IL18 [57]. A subsequent study elucidated that sorafenib administration led to the pyroptosis of macrophages, which then activated NK cells and resulted in HCC cell death [58]. Consistently, decreased expression of the major histocompatibility complex class I has been reported in the sorafenib-treated HCC cells. This chance hinders recognition by T cells and favours the NK cells-mediated response [58]. Moreover, the androgen receptor was documented to suppress IL-12A expression and inhibit the cytotoxic effect of NK cells against cancer cells. Sorafenib could enhance IL-12A signals through androgen receptor inhibition [59]. These results indicated that the therapeutic efficacy partially depended on the crosstalk between sorafenib and NK cells and provide a rationale to improve drug efficacy via combing NK cell-based therapy with sorafenib. In contrast, some studies reported that sorafenib could inhibit the proliferation and function of NK cells [60]. For instance, sorafenib was stated to dose- and time-dependently decrease the number of NK cells, downregulate the stimulatory receptor CD69 in NK cells, inhibit NK cell proliferation, decrease NK cell cytotoxicity by suppressing the pERK1/2 pathway and blocking the PI3K/AKT pathway [61–63]. Therefore, the optimized time and dosage must be determined when combining NK cell therapy with sorafenib.

The immunomodulatory effect of sorafenib on TAMs is quite controversial. Accumulating evidence has strengthened the idea that TAMs are generally classified into the pro-inflammatory subset (M1) and the anti-inflammatory subset (M2). Macrophage polarization is the activation state of macrophages tightly regulated by the TME and metabolism [64–66]. The drug efficacy of sorafenib was shown to partially depend on its effect on TAMs through miR-101 inhibition, reduced VEGF expression, macrophage sensitization, and pyroptosis induction [57, 67, 68]. Transfection of miR-101 has been shown to reduce dual specificity phosphatase 1 expression and cause subsequent down-regulation of ERK1/2, p38, and JNK. Sorafenib was reported to inhibit miR-101 expression and reduce the secretion of TGF-β and CD206 in M2, indicating a shift to the M1 polarized state [67]. As aforesaid, sorafenib could also sensitize macrophages and induce pyroptosis of macrophages and pro-inflammatory cytokines (IL6, TNF-α, and IL12) secretion, leading to subsequent NK cell activation [57, 58]. However, an in vitro study revealed that sorafenib could increase PD-L1 expression in tumour stroma and induce M2 accumulation through upregulating stromal cell-derived 1 alpha (SDF1α) [33]. Sorafenib was subsequently shown to induce M2 polarization in vivo but not in vitro. IFN-α was also proposed to shift the M2-like polarization of TAMs [69]. Additionally, sorafenib was shown to increase the infiltration of macrophages and Treg cells through the HIF-α/NF-κB pathway [70]. A review article compared the results of different studies examining the impact of sorafenib on macrophage in the TME. It raised the consideration that the unclear functional definition of TAMs, different dosages of sorafenib, and the impact of in vivo and in vitro studies may be responsible for discrepancy, as macrophage may experience dynamic changes after exposure to cytokines [23, 71, 72]. Therefore, the precent review described the effect of sorafenib on TAM based on the dosage and the model used (Table 2). In contrast to the results of the published review mentioned above, the cancer cell type is restricted to HCC. These results mainly suggested that sorafenib promoted the pro-inflammatory cytokines secretion in macrophages, which partially explained its antitumour effect, but its effect on altering polarization remained controversial.

**Table 2:** Current results for the effect of sorafenib on TAMs in HCC

Model	Dosage	Conclusion	References
Murine HCC cell line HCA-1	50 mg/kg	Sorafenib induces polarization towards a pro-immunosuppressive environment and M2 accumulation	[33]
Macrophages from human HCC tissue	1.2 μg/ml	Sorafenib revert the immunosuppressive effect of TAMs	[57]
In vivo: iAST mice	90 mg/kg 60 mg/kg 30 mg/kg 10 mg/kg	Sorafenib upregulates proinflammatory cytokine secretion and induce pyroptosis in macrophage	[58]
Monocyte-derived M1 and M2 macrophage cultures	1.2 μg/ml 2.5 μg/ml 5.0 μg/ml	Sorafenib revert the alternative macrophage polarization	[67]
In vitro: CSF-1 (M1) or GMCSF (M2) maturated monocyte-derived macrophages In vivo: patients with confirmed HCC	In vitro*:* 1.2 μg/ml 2.5 μg/ml 5.0 μg/ml In vivo: 800 mg/day	Sorafenib revert the alternative macrophage polarization, shifting the phenotype towards the M1-polarized state in vitro, and partially inhibits macrophage activation in vivo	[68]
C57BL/6 mice bearing tumour of approximately 50 mm^3^ in volume	30 mg/kg	Sorafenib induces the macrophage polarization in vivo but not in vitro	[69]

The interplay between TANs and sorafenib is also worth noting. TANs have been suggested to exert protumour effect, and TANs recruit Treg cells and macrophages via CCL2 and CCL17 and their receptors. Sorafenib was shown to increase the numbers of TANs and the levels of CCL2 and CCL17 in a mouse HCC model [73]. Regarding Treg cells, on the one hand, recruitment of Treg cells was observed through an increased number of TANs and increased expression of CCL2 and CCL17 [73]. On the other hand, other studies conducted in HCC murine models revealed that sorafenib inhibited the recruitment of Treg cells in HCC [74, 75]. In the case of T cells, although a study showed an increase of CD4+ and CD8+ T cell infiltration in murine models in other malignancies after treatment with sorafenib, a recent study reported that the infiltration of CD4+ and CD8+ T-cells remained constant in HCC models [58, 76]. This finding is consistent with the observation that T cells play no role in the therapeutic effect of sorafenib on HCC [57].

Despite the immunomodulatory effect, sorafenib also induces TME remodelling through interacting with hepatic stellate cells (HSCs). HSCs are mainly responsible for extracellular matrix production and deposition, and have been reported to stimulate HCC cell proliferation and vascularization [77]. In human HSCs treated with different concentrations of sorafenib, sorafenib was shown to dosage dependently inhibit ERK1/ERK2 and Akt phosphorylation, suppress the PDGF signalling pathway, and down-regulated the secretion of PDGF-BB and TGF-β1 from HSCs. As a result, the invasion and proliferation of HCC cells were inhibited [78]. Nonetheless, after sorafenib administration in mouse model of HCC, the survival of HSCs increased through the upregulation of stromal-derived factor 1 alpha (SDF-1α) expression in both tumour and stromal cells and activation of the SDF-1α/C-X-C receptor type 4 (CXCR4) pathway [79]. Meanwhile, MAPK activation was reported in HSCs and promoted the myofibroblast differentiation [80]. More evidence is still needed to verify the effect.

**Fig. 4:**
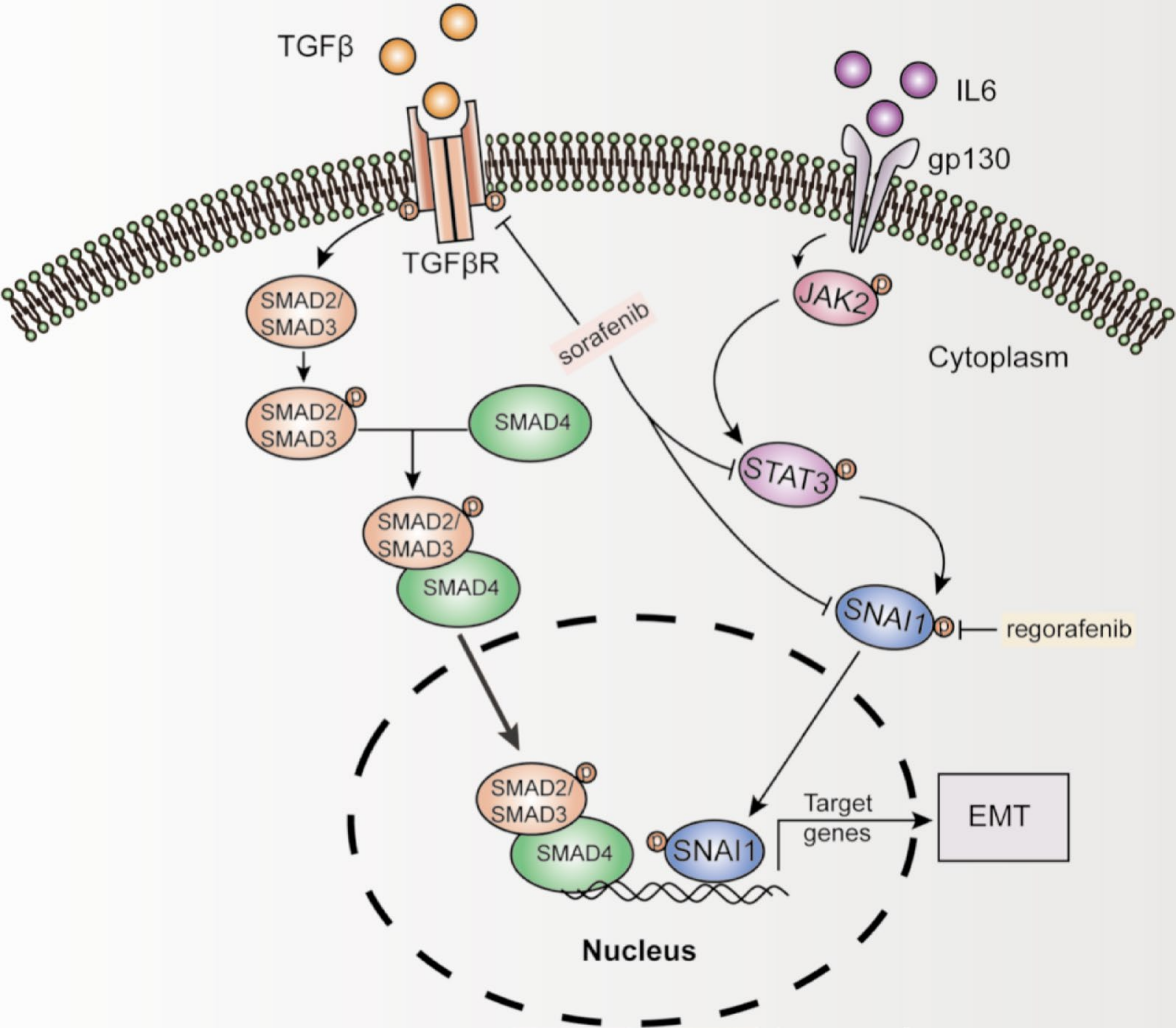
Effect of sorafenib and regorafenib on the EMT in HCC. Several signaling pathways contribute to EMT. Among them, sorafenib was shown to degrade the transforming growth-factor beta receptor 2 (TGFβ2), which further activates SMAD2 and SMAD3 and subsequently induces the EMT. Sorafenib was also suggested to inhibit STAT3 and SANI1 expression. Regorafenib has been shown to obstruct the progression of the EMT through STAT3 inhibition and a decrease in the expression of ID1 and SNAI1 in HCC

It is well established that the cellular interaction within the TME plays a pivotal role in the EMT. The EMT, the process characterized by a loss of apical-basal polarity and cell–cell adhesion in epithelial cells and a transition to mesenchymal cells, plays a pivotal role in malignant progression and drug resistance [81]. It is triggered by mitogen-activated protein kinase (MAPK), mTOR, and Wnt signalling [82]. Snail homologue 1 (SNAI1) and snail homologue 2 (SNAI2) have also been recognized as key inducers of the EMT, which predict a poor prognosis of HCC [83]. TGF-β, FGF, IL-6, hepatocyte growth factor, insulin-like growth factor-1, and epidermal growth factor derived from CAFs and immune cells in the TME were also identified as EMT inducers [84–86]. TGF-β, hepatocyte growth factor, epidermal growth factor, and FGF was shown to induce SNAIL production [84, 87, 88]. Specifically, TGF-β contributes to sorafenib resistance in HCC [89]. Sorafenib was postulated to inhibit the TGF-mediated EMT possibly via inhibition of TGF-β and MAPK signalling, and SNAI1 expression in HCC, thus inducing antitumour effects [83, 90]. Blockade of signal transducer and activator of transcription 3 (STAT3) by sorafenib was also reported (Fig. 4) [91, 92]. According to one study, the inhibition of TGF-β was achieved by inducing degradation of cell-surface TβR-II and caveolae/lipid raft-mediated internalization [93]. The underlying mechanism of the induction of the EMT during resistance acquisition following sorafenib administration was also suspected to be associated with abnormal miRNA expression [94]. However, long-term exposure to sorafenib was shown to cause drug resistance with EMT [95]. Sorafenib was also shown to promote the EMT, upregulate snail expression, and activate PI3K/AKT signalling in vitro [96]. Moreover, sorafenib increased the IL-6 expression in HCC cells and promoted metastasis and EMT progression in HCC cells. Knockdown of IL-6 could significantly decrease sorafenib resistance in HCC cells [97]. IL-6 is one of the pro-inflammatory cytokines secreted by TAMs, and sorafenib was shown to induce IL-6 secretion by TAMs [57, 58]. A reasonable hypothesis is that the regulation of sorafenib on TAM contributes to induction of EMT through IL-6 expression. The results mentioned above may suggest that the impact of sorafenib on EMT experience changes with different regimens and dosing. Current evidence has suggested the possibility of overcoming sorafenib sensitivity by targeting processes involved in the EMT.

An important mechanism accounting for the resistance-inducing effect described above is hypoxia caused by decreased vascularization. Hypoxia-mediated TME remodelling usually depends on HIF-driven transcriptional responses [98]. The HIF family comprises of HIF-1, HIF-2, and HIF-3. The HIF-α subunits of HIF-1 and HIF-2 are oxygen-sensitive and correlate with tumour progression [99]. Hypoxia was shown to correlate with the EMT and is related to the formation of the immunosuppressive microenvironment through not only homing bone marrow-derived cells (BMDCs), TAMs, and Tregs but also shaping and inducing specific macrophage phenotypes. Specifically, hypoxia was shown to induce M2 polarization and educate PBNs into TANs to promote malignant progression [33, 100, 101]. Several studies concluded that sorafenib could induce HIF-α accumulation and cause subsequent activation of resistant-inducing pathways. NF-κB activation and increased stromal-derived factor 1 alpha (SDF-1α) expression were also observed [79]. Further research based on photoacoustic imaging showed that sorafenib induced a decrease in oxygen saturation and an increase in HIF-α levels [102]. Although a previous study demonstrated that HIF-α was dose-dependently inhibited by sorafenib, recent evidence supports the hypothesis that sorafenib induces HIF-α accumulation, which partially accounts for the anti-angiogenic effect and resistance-inducing mechanism of sorafenib [79, 102, 103].

### Lenvatinib

**Fig. 5:**
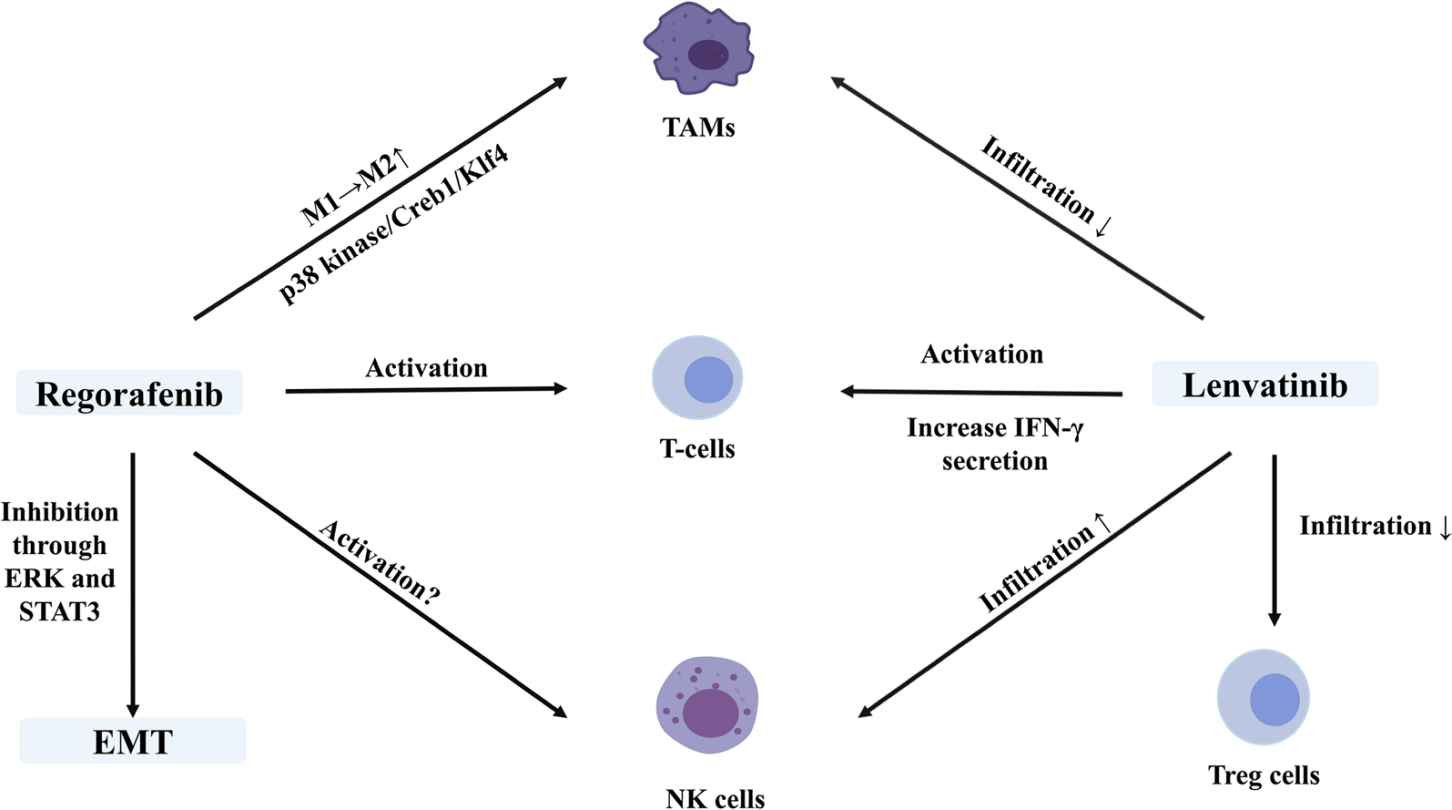
Modulatory effects of regorafenib and lenvatinib on the HCC TME. Regorafenib was shown to revert M2 to M1 polarization, activate TANs, and inhibit EMT, while its effect on activating NK cells required more evidence. Lenvatinib decreased the population of TAMs in HCC while increasing the populations of NK, and cytotoxic T cells

Lenvatinib is approved as a first-line TKI based on a phase 3 clinical trial conducted among 954 patients with unresectable HCC. With a Median OS of 13.6 months for patients in the lenvatinib arm (compared to 12.3 months for patients in the sorafenib arm) (hazard ratio 0.92, 95% confidence intervals 0.79–1.06), lenvatinib was determined to provide an overall survival benefit that is not inferior to sorafenib in patients with HCC (NCT01761266) [18]. The most common adverse effects were hypertension, diarrhea, appetite decline, and loss of weight in patients treated with lenvatinib [18]. Similar to sorafenib, lenvatinib is also a multitarget TKI that targets VEGFR1-3, TIE2, KIT, RET, RAF-1, BRAF, BRAFV600E, PDGFR, and FGFR1-4 [104–107]. Compared to sorafenib, lenvatinib exerts a more potent effect on VEGFRs and FGFRs, the inhibition of which might improve immunity due to the immunosuppressive role of VEGFRs and FGFRs [108]. Hence, the current investigations of the effect of lenvatinib on the TME in HCC mainly focus on the immune microenvironment (Fig. 5).

In addition to its antiangiogenic effect, the antitumour effect of lenvatinib was observed to partially depend on the existence of CD8+ T cells, TAM modulation, a reduced Treg proportion, and increased IFN-γ secretion by cytotoxic T cells in murine HCC models [108, 109]. Notably, a study conducted in a murine HCC model reported that CD8+ T cell depletion significantly decreased the potency of lenvatinib, but induced no significant change in the efficacy of sorafenib [108]. Moreover, lenvatinib was also observed to decrease the fractions of macrophage and monocyte populations but increase CD8+ T cell populations [108]. A recent study further revealed that the antitumour effect of lenvatinib in HCC mainly depended on its antiangiogenic effect and its modulation of TAMs. Lenvatinib was shown to decrease the population of TAMs in HCC while increasing the populations of NK and cytotoxic cells in a murine HCC BNL model [109]. Additionally, IFN-γ and granzyme B secretion from cytotoxic T cells increased [109]. In consistence, a study obtaining peripheral immune cells and cytokine profiles of patients with HCC reported that the administration of lenvatinib reduced the numbers of T helper cells and Treg cells, significantly increased the numbers of cytotoxic T cells, increased IL-2, IFN-γ, and IL-5 levels, and decreased IL-6, IL-10, TNF-α, and TNF-β levels, suggesting that further investigations into the immunomodulatory effect of lenvatinib on TME are needed [110].

Interestingly, overexpression of PD-1 induced exhaustion of activated CD8+ cells, and inhibition of PD-1 has been approved as a third-line therapy for advanced HCC in 2017 [111, 112]. The reliance of lenvatinib on the existence of CD8+ T cells has shed light on combining pembrolizumab or nivolumab with lenvatinib [108, 113]. Several animal experiments revealed that the combination of lenvatinib and anti-PD-1 therapy amplified the antitumour effect on HCC, and amplification was presumed to be achieved by exerting immunomodulation through a reduction in TAM infiltration, synergistic modulation on T cells, reversion of immunosuppressive effect caused by anti-PD-1 therapy, and vascular normalization [109, 114, 115]. Long-term administration of PD-1 in patients was reported to increase of VEGF and FGF expression, and lenvatinib exerts a more potent effect on VEGFRs and FGFRs compared to sorafenib as mentioned [108, 114]. In this context, reversion of the immunosuppressive effect caused by anti-PD-1 was proposed to be achieved by upregulating PD-1, cytotoxic T lymphocyte-associated protein-4, and TIM-3 on T cells, downregulating IFN-γ and granzyme B, and inhibiting T cell cytotoxicity [114]. The immunomodulation exerted by the combination of lenvatinib and anti-PD-1 was recently proven to be associated with TGF-β inhibition [115].

Based on these results, clinical trials combing anti-PD-1 therapy with lenvatinib are ongoing. In patients with advanced gastric cancer, a single arm, phase 2 trial reported an ORR of 69% (NCT03609359), indicating potential in HCC therapy [116]. For patients with unresectable HCC, an open-label, multicentre, phase Ib trial has shown that combining lenvatinib with pembrolizumab provided promising antitumour activity with a tolerable safety profile. The median overall survival was 22 months and the objective response rate was reported to be 44.8% in the group receiving lenvatinib and pembrolizumab (compared with 24.1% for patients in the lenvatinib arm of the REFLECT trial) (NCT03006926) [117]. A multicentre, double-blind, phase 3 trial that further investigates this combination is ongoing (NCT03713593).

### Regorafenib

Regorafenib, a recently approved second-line TKI for patients with HCC who previously received sorafenib, has been proven to significantly prolong the time to progression in a clinical trial engaging 573 patients with HCC progression following sorafenib treatment (NCT01774344) [19]. The adverse events were reported to be hypertension, hand-foot skin reaction, fatigue, and diarrhoea [19]. With one hydrogen atom replaced by a fluorine atom, regorafenib was reported to have higher potency when compared to sorafenib [118, 119]. Targeting VEGFR, FGFR, PDGFRA, KIT, and RET, regorafenib also inhibits the RAF/MEK/ERK pathway [120]. ERK and STAT3 signalling are EMT-inducing pathways in HCC. The anti-HCC effect of regorafenib was shown to be partially induced by p-STAT3-related signaling inhibition through apoptosis [121]. Compared to sorafenib, regorafenib was also demonstrated to exhibit more potent efficacy in STAT3 inhibition through a mechanism mediated by SHP-1 [71].

Regorafenib was shown to modulate macrophage polarization, induce T cell activation, and mediate NK cell function, which enhanced its antitumour effects (Fig. 5). Reversion of M2 polarization in multiple syngeneic liver cancer models was achieved by suppressing the p38 kinase/Creb1/Klf4 axis. Interestingly, the efficacy of adoptively transferred antigen-specific T cells increased after treatment of regorafenib, suggesting the potential for combination therapy [122]. Another recent finding demonstrated that regorafenib treatment inhibited STAT3 and mediated a subsequent increase in CXCL10 expression at both the transcript and protein levels in the murine model and human peripheral blood. CXCL10 is a ligand for CXCR3 that is expressed on tumour-infiltrating lymphocytes and mediates CD8 T cell infiltration within tumour [123]. Moreover, the NK cell function is mediated by CD24 level, which is controlled by p-STAT3. As regorafenib downregulates the p-STAT3 signalling as mentioned above, it might enhance the antitumour effect of NK cells [124]. Regorafenib was also shown to inhibit the expression of MMP 9 as sorafenib did in liver cancer murine model, and thus inhibiting the shedding of MICA [125]. Additionally, in melanoma, the antitumour effect of NK cells is limited by the expression of HLA Class I. Regorafenib was reported to suppress HLA Class I-mediated tumour progression, suggesting the possibility of a similar effect on HCC [126, 127]. Accumulating evidence suggested that regorafenib augments the efficacy of NK cells, but further studies are still needed to verify this effect.

Apart from the immune microenvironment, regorafenib has been shown to obstruct the progression of the EMT by inhibiting of ERK and STAT3 (Fig. 4) [128]. Another study proposed that regorafenib inhibits the EMT by suppressing inhibitor of differentiation 1 (ID1) expression, which downregulates SNAI1 and promotes the EMT [129]. Research in colorectal cancer has described the inhibitory effect of regorafenib on the TGF-β1-induced EMT via enhancement of SHP-1 activity [130]. Interestingly, the effect of regorafenib on the EMT counteracts with sorafenib resistance induced by hepatocyte growth factor, suggesting the rationale for its application in sorafenib resistant patients [131].

### Cabozantinib

Approved by FDA based on a clinical trial, cabozantinib achieved a median OS of 10.2 months in 470 patients with HCC (compared to 8 months in 237 patients who received the placebo) (hazard ratio 0.76, 95% confidence intervals 0.36–0.52; *P* = 0.0005) (NCT01908426) [20]. The side effects were reported to be hand-foot skin reaction, hypertension, increased level of aspartate aminotransferase, fatigue, and diarrohea [20]. Cabozantinib is a second-line TKI for HCC that targets VEGFR1-3, MET, AXL, and c-KIT. It was reported to exert its antitumour effect mainly by inhibiting VEGFR and cellular-mesenchymal epithelial transition factor (c-MET). C-MET was proven to induce hepatocarcinoma initiation in mice in cooperation with Wnt/β-catenin or Akt/mTOR cascades [132, 133].

Cabozantinib was proposed to exert an immunomodulatory effect by stimulating the neutrophil infiltration and reducing the macrophage infiltration [134, 135]. Previous results from a PTEN/p53-deficient murine prostatic carcinoma model showed that the administration of cabozantinib amplified the secretion of neutrophil chemotactic factors, including CXCL12 and HMGB1, leading to neutrophil infiltration [134]. It was also observed to downregulate M1 macrophages to prevent bone metastasis in prostate cancer cells, whereas it potentiated the growth of prostate carcinoma-associated fibroblasts at the same time [136]. In MC-38-CEA tumour cells derived from murine colon adenocarcinoma, cabozantinib was observed to alter the cell subception in the immune microenvironment, reducing macrophage infiltration [135]. Although evidence has accumulated for other malignancies, a recent study of HCC indicated no significant alteration in immune cells was observed in both c-Met/β-catenin or Akt/c-Met murine models. Interestingly, the combination of cabozantinib with a PD-1 inhibitor increased the numbers of spleen CD3+ CD8+ and CD3+ CD4+ PD-1+ cells in Akt/c-Met mouse [137]. Notably, cabozantinib was shown to have a limited effect on the macrophages, tumour-infiltrating T cells, CAFs, and fibrosis, which differs from the hypothesis proposed before the research [137]. The expression of PD-1 remained unchanged after cabozantinib treatment, and combining cabozantinib with an anti-PD-L1 antibody showed no increase in survival benefit in a nonclinical study [137]. However, a recent clinical trial reported that the combination of nivolumab, ipilimumab, and cabozantinib generated an ORR of 26% for patients with HCC (ORR = 17% in the nivolumab + cabozantinib arm), but the adverse events increased [138].

## Combination therapy based on TKI-induced TME remodelling: current state and future perspectives in advanced HCC

Although liver transplantation and liver regeneration are actively studied, systematic therapy is still commonly used in treating advanced HCC [139, 140]. TKIs remain the backbone for systematic treatment for advanced HCC, but toxicity, resistance, and relapse continuously hinder the clinical use of TKIs in HCC. TKIs are currently gradually being replaced by the combination of atezolizumab and bevacizumab. Accumulating evidence has indicated the promising future of remodelling the TME with combination therapy to overcome the TKI resistance of HCC. For instance, adding sorafenib to MnO_2_ with nanoparticles to alleviate hypoxia in the TME effectively overcomes sorafenib resistance [141]. Moreover, treatments targeting the EMT process plus sorafenib were also reported to enhance the clinical response compared to sorafenib alone [142]. SNAI1 and the proteins that stabilize it were shown to be upregulated in sorafenib-resistant HCC cells. Knockdown of SNAI1 and the proteins stabilizing SNAI1 restore sensitivity to sorafenib [143]. Combination therapy of sorafenib and strategies blocking the EMT, including SB431542 (TGF-β mediated EMT), valproic acid (anti-epileptic drug), curcumin, thioredoxin inhibitors, urinastatin, anti-CDK1, ADAM17 inhibitors, C2-ceramide, ATM-inhibitors, anti-human IL-17A monoclonal antibody, destruxin B, snail signalling inhibitors or IL‑6/STAT3 signalling inhibitors have shown potential as novel strategies to combat drug resistance through EMT downregulation [143–155]. Likewise, the combination of lenvatinib with c-MET inhibitors or histone deacetylase was also suggested to downregulate the EMT and improve the systematic therapeutic effect [156, 157]. Furthermore, a recent study conducted in the human cell line observed that the combination of the PI3K/mTOR inhibitor BEZ235 with regorafenib inhibited the expression of EMT-related proteins namely Slug, MMP-9, MMP-2, and Vimentin [158]. The combination of regorafenib with Pin1 inhibitors that interact with EMT regulators has also been suggested as a potential strategy [159]. Nonetheless, randomized clinical trials are still needed to test the therapeutic efficacy and safety profile.

TKIs induce immunomodulatory effects, and no overlap has been observed between the major toxicity profiles of TKIs and ICIs. Thus, a great rationale exists for the combination of TKIs and ICIs [160]. Theoretically, the efficacy of antiangiogenic treatment might also be augmented by ICI application through the relief of the immunosuppressive microenvironment, and TKIs may complement ICI regarding the low ORR of ICIs [161, 162]. Clinical trials conducted in patients with other malignancies, including metastatic renal cell carcinoma and melanoma, have shown a manageable toxic profile for the combination of TKIs and ICIs [163, 164]. Exciting results have been generated in the other malignancies. A phase 3 trial of renal cell carcinoma showed that the combination of TKI (axitinib) with pembrolizumab significantly prolongs overall survival and progression-free survival compared to sunitinib alone [165]. Combining lenvatinib with anti-PD1 as a treatment for advanced gastric cancer has also shown satisfactory results in a phase 2 clinical trial [166].

Regarding HCC, the outcomes of clinical trials combining TKIs with immunotherapy differ for different TKIs. Clinical trials that combined 200 mg sorafenib bid (days 1–28) with 2.5 mg/kg bevacizumab (days 1 and 15) were discontinued at phase 2 due to low efficacy and excessive toxicity (NCT00867321) [167]. The immunosuppressive effect of sorafenib and the comparison of the profile between sorafenib and lenvatinib might partially explain the failure of the combination of sorafenib with PD-L1 [108]. In addition, unlike lenvatinib, which partially depends on T cells, the therapeutic effect of sorafenib was shown to be mainly mediated by macrophage and NK cell responses [57]. The interaction between NK cells and sorafenib may provide the rationale for the further combination of NK cell-based therapy with sorafenib (Fig. 3). Agents developed to target MICA and MICA B shedding and increasing the NKG2D ligands expression include matrix metalloproteases, antibodies targeting the MICA/B α3 domain, and histone deacetylase inhibitors [168, 169]. NK cell therapy ongoing trials for HCC includes allogenic NK therapy (NCT04162158), adoptive transfer of iNKT cells (NCT04011033), FATE-NK100 (NCT03319459), and FT500 (NCT03841110, NCT04106167). Their combination with sorafenib still needs verification. Targeting the androgen receptor that is involved in the regulation of sorafenib on the NK cells might also act as a novel therapeutic strategy in synergistic with sorafenib.

For the other TKIs listed, recent research has shown a promising future for combination strategies. The combination of ICIs with lenvatinib or cabozantinib in HCC has shown promising results [116, 138]. Currently, many ongoing clinical trials focuses on the combination of lenvatinib with other anti-PD-1 agents including toripalimab (NCT04523493), camrelizumab (NCT04443309), and HX008 (NCT04741165), and a summary of ongoing trials up to 2020 has been reported by Huang et al. [170]. TME remodelling induced by lenvatinib has provided the rationale for its combination with immunotherapy, while the results for cabozantinib remain unclear. Trials investigating the combination of immunotherapy with other TKIs including anlotinib (NCT04172571) and apatinib (NCT03764293) are also ongoing. Evidence for TME remodeling by other TKIs is still limited. Meanwhile, the fact that regorafenib was shown to enhance the efficacy of adoptively transferred T cells is also worth noting for future progress.

## Conclusion

This review outlined the modulation of the TME of HCC by TKIs. The microenvironment of HCC is characterized by immunosuppressive microenvironment and profound extracellular matrix remolding. Currently, four TKIs are approved by FDA as HCC therapy, the other TKIs ongoing trials are also described in the review. Based on current evidence, the effect of sorafenib on the TME of HCC remains controversial. Generally, sorafenib was shown to activate NK cells by regulating the shedding of MICA, interacting with macrophages, and inhibiting androgen receptors. Sorafenib was also shown to interact with TAMs, cytotoxic T cells, Tregs, and HSCs in HCC. Lenvatinib, regorafenib, and cabozantinib also exert immunomodulatory effects, contributing to the rationale for combining lenvatinib and cabozantinib with anti-PD1 as treatment for HCC. Additionally, sorafenib was shown to inhibit the TGF-mediated EMT, while regorafenib obstructed the EMT through ERK and STAT3. Based on the understanding of the effect of TKIs on the TME, combination therapy based on TKI-induced TME remodeling is worth further investigation.

We provide a summary of TME-induced remodelling specifically in HCC and thus summarize the rationale and potential target for combination therapy in HCC. However, insufficient evidence is available for the modulatory effects of cabozantinib, regorafenib, and lenvatinib. Further investigations of the following aspect are needed: (1) Exploration of the unidentified effects and underlying mechanism by which TKIs modulate HCC. More specifically, the roles of HSCs and endothelial cells in the TME modulation process have not been fully investigated. As current research has proven the efficacy of combining cabozantinib and anti-PD1, further investigations of the immunomodulatory effect of cabozantinib are also suggested. (2) Identification and verification of strategies designed to overcome resistance and combination therapy related to the TME of HCC. Potential targets of EMT in combination with TKIs have been listed, while their translation to the clinic requires more effort. Additionally, the combination of NK cell therapy with sorafenib must be validated in clinical trials and determine the optimal dosage and time. With the success of the combination of lenvatinib and anti-PD1, investigations examining cabozantinib and other TKIs, including apatinib, anolotinib, and those currently being analyzed in clinical trials, should be emphasized.

## Supplementary Information

**Additional file 1. **The flowchart of searching strategies.

## Data Availability

Not applicable.

## Abbreviations

HCC: Hepatocellular carcinoma
TKIs: Tyrosine kinase inhibitors
FDA: Food and Drug Administration
VEGFR: Vascular endothelial growth factor receptor
TME: Tumour microenvironment
VEGF: Vascular endothelial growth factor
PDGF: Platelet-derived growth factor
FGF: Fibroblast growth factor
TGFs: Transforming growth factors
HIF-1: Hypoxia-inducible factor-1
NK: Natural killer
TANs: Tumour-associated neutrophils
Tregs: Regulatory T cells
Anti-PD-L1: Anti-programmed cell death protein 1
ORR: Objective response rate
OS: Overall survival
ICI: Immune checkpoint inhibitor
TAMs: Tumour-associated macrophages
MDSCs: Myeloid-derived suppressive cells
CAFs: Cancer-associated fibroblasts
MICA: Major histocompatibility complex class I-related chain A
ADAM: A disintegrin and metalloproteinase
MMP: Matrix metalloprotease
INF: Interferon
SDF1α: Stromal cell-derived factor1 alpha
MAPK: Mitogen-activated protein kinase
SNAI1: Snail homologue 1
SNAI2: Snail homologue 2
STAT3: Signal transducer and activator of transcription 3
HSCs: Hepatic stellate cells
BMDCs: Bone marrow-derived cells
STAT3: Signal transducer and activator of transcription 3
c-MET: Cellular-mesenchymal epithelial transition factor
